# Supplementary material for: Speeding up iterative applications of the BUILD supertree algorithm
Source: PeerJ. 2024 Jan 2;12:e16624. doi: 10.7717/peerj.16624 (PMC10768670; doi:10.7717/peerj.16624)
Supplement: Supplemental Information 8 [file peerj-12-16624-s008.pdf]

A PROOF OF INVARIANTS

A.1 At least one of S or O is empty

When BuildIncA″ is first called, O has no splits, so the invariant holds.

The RemoveIrrelevantSplits step can alter S and O in two ways. First, if O contains a solution S′ with the same taxon set as S, then we can replace S with S′ and clear O. Second, if one of the sub-solutions in O is punctured, we remove it from O and add its child solutions to O, but S remains empty. In both of these cases, the invariant is preserved. Further steps inside BuildIncA″ do not modify S or O.

When BuildIncA″ is recursively called on an unmodified component, O will be empty. When BuildIncA″ is called on a modified component or a new component, S will be empty. So, this invariant is preserved in recursive calls to BuildIncA″.

Therefore, since the invariant is initially true, and is preserved within BuildIncA″ and at recursive calls, it holds by induction.

A.2 Original solutions in O have a taxon set inside S.T

O is empty when BuildIncA″ is first called. When C.O is initially filled by merging two components, all the original solutions in C.O are for original components that are subsets of the new component. Therefore, when calling BuildIncA″(C.S, C.ΔΣ, C.O), all the solutions in O=C.O will have a taxon set inside S.T.

When RemoveIrrelevantSplits replaces a punctured solution with its child solutions, the child solutions remain inside the taxon set of the current problem, since they are contained within their parent taxon set, which was inside the taxon set of the current problem. QED.

A.3 Original solutions in O have non-overlapping taxon sets

O is empty when BuildIncA″ is first called. When O is initially filled by merging a component, all the solutions in O must have non-overlapping taxon sets, since they are solutions for different equivalence classes on the same level.

When RemoveIrrelevantSplits replaces a punctured solution by its child solutions, it preserves this property, since the taxon sets of the child solutions are non-overlapping, and are contained within the taxon set of their removed parent. Thus the solutions in O will always have non-overlapping taxon sets.

B PROPERTIES OF SOLUTION S

B.1 Extracting the tree from a Solution

If the full Build algorithm succeeds, a tree can be extracted from the Solution object. A Solution object S maps to a node *n* in the tree. The node's children consist of either (i) internal nodes (one for each component in S.$\mathscr{C}$) or (ii) leaves (one for each trivial component; *i.e.* taxon identifier for which S.M is a Null reference). The original Solution object created in Build corresponds to the root of the tree.

B.2 Extracting the splits Σ from a Solution

Each Solution object S is created by performing BuildA on a set of splits Σ. If BuildA(S,Σ) succeeds, then after it is complete S will contain the splits Σ. However, only the splits S.I are *directly* contained in S. The remaining splits of Σ that are not stored in S.I are contained in the child solutions of S (and their descendants).

We can recover the set of splits in S as follows:

$$\Sigma(S) = \mathtt{S.I} \cup \left(\bigcup_{C \in S.\mathscr{C}} \Sigma(C.S) \right). \tag{1}$$

A solution is considered "empty" if it contains no splits. This can only happen if both S.I and S.$\mathscr{C}$ are empty. It is possible for S.$\mathscr{C}$ to be empty while S.I is non-empty, so both fields must be used.

B.3 The relationship between S.T and $\Sigma(S)$

Additionally, if S is a solution to some component C, then the taxon set S.T is determined by the splits Σ(S):

$$\mathtt{S.T} = \bigcup_{\sigma \in \Sigma(S)} \sigma_1 \tag{2}$$

Addionally the taxa in `S.T` form a connected component that is connected *only* through edges corresponding to splits in $\Sigma($`S`$)$.

The only exception to equation (2) is the top level `Solution`. The top level taxon set is directly initialized by the user and does not correspond to a component that was identified by running BUILDA, so there may be taxa in T that are not mentioned in Σ. These taxa will connect directly to the root since they are not in the include group of any split. However, for `Solution` objects below the top level, equation (2) holds.
