# Supplementary material for: Speeding up iterative applications of the BUILD supertree algorithm
Source: PeerJ. 2024 Jan 2;12:e16624. doi: 10.7717/peerj.16624 (PMC10768670; doi:10.7717/peerj.16624)
Supplement: Supplemental Information 7 — A snapshot of otcetera code base with SHA 2287cefc4d12d328c4d9a4272b10aa25b38abbcd from https://github.com/OpenTreeOfLife/otcetera. The algorithms described in this article are implemented in the otc-solve-subproblem tool. [file peerj-12-16624-s007.gz › otcetera/test/tnrs/data/taxonomy-tree/out1.0/cleaned_phylo/index.html]

Propinquity output documentation. The top-level docs are here.

The *cleaned\_phylo* contains the results of pruning problematically
mapped tips from phylogenetic inputs. Full documentation of the procedure and
artifacts can be found in the
README. Note that trees in this directory may have tips that are mapped
to higher (non-terminal) taxa in the OpenTreeTaxonomy.

**Next step:** The next step in phylogeny processing is to choose
exemplars for tips that are mapped to higher taxa (see
../exemplified\_phylo/index.html).
First, though, we pre-process the Open Tree Taxonomy (see
../cleaned\_ott/index.html).

**Results:** There were 2
nonempty trees after cleaning. The files of interest in this directory are:

| JSON log of pruning | pruned newick |
| --- | --- |
| xx\_1@1.json | xx\_1@1.tre |
| xx\_2@1.json | xx\_2@1.tre |
