# Supplementary material for: Speeding up iterative applications of the BUILD supertree algorithm
Source: PeerJ. 2024 Jan 2;12:e16624. doi: 10.7717/peerj.16624 (PMC10768670; doi:10.7717/peerj.16624)
Supplement: Supplemental Information 7 — A snapshot of otcetera code base with SHA 2287cefc4d12d328c4d9a4272b10aa25b38abbcd from https://github.com/OpenTreeOfLife/otcetera. The algorithms described in this article are implemented in the otc-solve-subproblem tool. [file peerj-12-16624-s007.gz › otcetera/test/tnrs/data/taxonomy-tree/out1.0/annotated_supertree/index.html]

Propinquity output documentation. The top-level docs are here.

The previous step (../labelled\_supertree/index.html) produced the final tree.
The *annotated\_supertree* directory just holds annoations of that tree

The files of interest in this directory are (click to download):

- ./annotations1.json: contains support and conflict statements for each edge in the synthesis tree
- ./annotations2.json: contains only metadata about the pipeline and the inputs used
- ./annotations.json: contains all of the information from annotations1 and annotations2

See README for discussion of these files and links to the description of the schema
