# Supplementary material for: Speeding up iterative applications of the BUILD supertree algorithm
Source: PeerJ. 2024 Jan 2;12:e16624. doi: 10.7717/peerj.16624 (PMC10768670; doi:10.7717/peerj.16624)
Supplement: Supplemental Information 7 — A snapshot of otcetera code base with SHA 2287cefc4d12d328c4d9a4272b10aa25b38abbcd from https://github.com/OpenTreeOfLife/otcetera. The algorithms described in this article are implemented in the otc-solve-subproblem tool. [file peerj-12-16624-s007.gz › otcetera/test/tnrs/data/taxonomy-tree/out1.0/subproblems/index.html]

Propinquity output documentation. The top-level docs are
here.

The *subproblems* directory holds the 2
subproblems. Each one is a slice of the full tree. They do not overlap.
The decomposition is produced
by identifying each higher taxon that is not contested by any single
phylogenetic input.
This taxon becomes the root of the subproblem (and is used in the file name to
identify the subproblem).
Many of the subproblems contain only trivial phylogenetic statements.
See the subproblems README for details on the procedure.

**Next step:** Solving the subproblems. See
../subproblem\_solutions/index.html
for details.

**Results:**
This page contains the list of subproblems, the
list of subproblems overlapping each input tree, and the
list of contested taxa.

**List of subproblems**

Each of the 2
subproblems is a list of trees (in newick
representation) with the order reflecting the ranking of the input trees.
The taxonomy overlaps with every subproblem.

Sorted by the number of phylogenetic inputs, the subproblems are:

| Subproblem | # inputs | # tips | Input Identifiers |
| --- | --- | --- | --- |
| ott5268475.tre | 3 | 2 | xx\_1@1.tre, xx\_2@1.tre, TAXONOMY |
| ott805080.tre | 3 | 2 | xx\_1@1.tre, xx\_2@1.tre, TAXONOMY |

**Overlap between trees and subproblems**

The list of input phylogenetic trees sorted by the number of subproblems that they overlap with is:

| Study+Tree key | # subproblems | Subproblem Identifiers |
| --- | --- | --- |
| xx\_2@1 | 2 | ott5268475, ott805080 |
| xx\_1@1 | 2 | ott5268475, ott805080 |

**Contested taxa**

The 0 contested taxa follow. For each taxon,
we list the input tree(s) that conflict with the taxon. For each tree,
the "parent nodes" are internal nodes each of which has
at least 1 child that belongs entirely to the taxon
and at least one descendant that does not belong in the taxon. If the OTT Id were
not contested, there would only be one internal node that fit those criterion, but
in contested taxa there are multiple.
