# Supplementary material for: Speeding up iterative applications of the BUILD supertree algorithm
Source: PeerJ. 2024 Jan 2;12:e16624. doi: 10.7717/peerj.16624 (PMC10768670; doi:10.7717/peerj.16624)
Supplement: Supplemental Information 7 — A snapshot of otcetera code base with SHA 2287cefc4d12d328c4d9a4272b10aa25b38abbcd from https://github.com/OpenTreeOfLife/otcetera. The algorithms described in this article are implemented in the otc-solve-subproblem tool. [file peerj-12-16624-s007.gz › otcetera/test/tnrs/data/taxonomy-tree/out1.0/phylo_input/index.html]

Propinquity output documentation. The top-level docs are here.

The *phylo\_input* directory holds the files that are used to extract the ranked list of
study+tree IDs for the phylogenetic inputs to the pipeline.

**Next step:** Copy the NexSON files as a snapshot.
See ../phylo\_snapshot/index.html
for details.

**Results:**

- collections.txt is simply a copy of the configuration setting that list the identifiers for the collections in order
- rank\_collection.json is a collection JSON that is the concatenation of the collections (which where found in the phylesystem shard
  described in the configuration documentation.
- study\_tree\_pairs.txt is list of the study ID + tree ID in a terse syntax that is appropriate for file names within this propinquity pipeline.
- These are the 2 trees used as inputs for this run (click any link to view a tree in a new window):
  1. tree 1 of study xx\_1
  2. tree 1 of study xx\_2
- index.json is a JSON representation of the summaries used when writing this page.
