# Supplementary material for: Speeding up iterative applications of the BUILD supertree algorithm
Source: PeerJ. 2024 Jan 2;12:e16624. doi: 10.7717/peerj.16624 (PMC10768670; doi:10.7717/peerj.16624)
Supplement: Supplemental Information 7 — A snapshot of otcetera code base with SHA 2287cefc4d12d328c4d9a4272b10aa25b38abbcd from https://github.com/OpenTreeOfLife/otcetera. The algorithms described in this article are implemented in the otc-solve-subproblem tool. [file peerj-12-16624-s007.gz › otcetera/test/tnrs/data/taxonomy-tree/out1.0/index.html]

The files in this directory (and its children) were produced using
propinquity,
a make-based pipeline for phylogenetic supertrees.

# Configuration

The configuration file for the propinquity run was at: `/home/mholder/opentree/cruft/fninp/out/config`

## Taxonomy

- OTT version: 0.0 (specifically version 0.0draft0)
- root taxon:
- The local copy of this version of OTT was at `/home/mholder/opentree/cruft/fninp/taxonomy`
- This version of OTT was pruned to removed taxa that are flagged with any of the following taxon flags:
  - barren
  - environmental
  - hidden
  - hybrid
  - inconsistent
  - major\_rank\_conflict
  - major\_rank\_conflict\_inherited
  - merged
  - not\_otu
  - viral
  - was\_container

## Phylogenetic inputs

The phylesystem shards for this run were located under `/home/mholder/opentree/versioned/phylesystem`

The collections shards for this run were located under `/home/mholder/opentree/versioned/phylesystem`. 0 collections were used, ranked in this order:


## Tools

### otcetera

- version: `unknown` and git SHA unknown
- compiled using version unknown of the boost C++ libraries

### peyotl

- the filepath of the root directory of peyotl was `/home/mholder/opentree/propinq_peyotl`
- version: `0.1.4dev` and git SHA 4c3e468dceb2866fe0e8ce9a276f1f661871b6fb

### propinquity

- git SHA 56259e16abe7deffb10da3df4f4715b81bc3c91d

## Processing of phylogenetic inputs

1. phylo\_input/index.html discusses
   the list of 2 input phylogenetic trees that were identified from the collections.
2. phylo\_snapshot/index.html links to
   the git SHA of these trees and copies of them as they existed when the supertree pipeline
   was run.
3. cleaned\_phylo/index.html describes the
   pruning of tips from input phylogenies due to mapping to problematic taxa. There were 2 nonempty trees after that cleaning.
4. exemplified\_phylo/index.html describes the choice of terminal taxa to exemplify those nodes that are mapped to higher taxa.

Logs for these steps are here (stdout) and here (stderr)

## Processing of taxonomic inputs

1. cleaned\_ott/index.html describes the removal of flagged taxa and pruning to the chosen root.
2. exemplified\_phylo/index.html describes how the choice of exemplars for the phylogenetic inputs also leads to a pruned taxonomy with these exemplars.

## Supertree algorithm

1. subproblems/index.html describes the decomposition of the full tree into adjacent subproblems.
2. subproblem\_solutions/index.html describes the solutions that result from each of these subproblems.
3. grafted\_solution/index.html describes piecing the subproblem solutions into a single solution for the exemplified taxa.
4. labelled\_supertree/index.html describes how the taxa pruned off in the exemplar step are reattached to produce a full supertree.

Logs for these steps are here (stdout) and here (stderr)

## Annotation of the supertree

See annotated\_supertree/index.html links to the annotations for that tree.

Logs for these steps are here (stdout) and here (stderr)

## Assessment of the pipeline

See assessments/index.html discusses what tests were
run to assess the supertree.

Logs for these steps are here (stdout) and here (stderr)

Logs for the HTML generation steps are here (stdout) and here (stderr)

An index for all of the logs is available here

## Final output

1. ./labelled\_supertree/labelled\_supertree.tre (the tree)
2. ./annotated\_supertree/annotations.json (the annotations file)
