# Supplementary material for: Speeding up iterative applications of the BUILD supertree algorithm
Source: PeerJ. 2024 Jan 2;12:e16624. doi: 10.7717/peerj.16624 (PMC10768670; doi:10.7717/peerj.16624)
Supplement: Supplemental Information 7 — A snapshot of otcetera code base with SHA 2287cefc4d12d328c4d9a4272b10aa25b38abbcd from https://github.com/OpenTreeOfLife/otcetera. The algorithms described in this article are implemented in the otc-solve-subproblem tool. [file peerj-12-16624-s007.gz › otcetera/test/tnrs/data/taxonomy-tree/out1.0/cleaned_ott/index.html]

Propinquity output documentation. The top-level docs are here.

The *cleaned\_ott* directory contains the results of pruning OTT
version 0.0draft0 so that the root is
and the taxa flagged with the following flags were removed:

- barren
- environmental
- hidden
- hybrid
- inconsistent
- major\_rank\_conflict
- major\_rank\_conflict\_inherited
- merged
- not\_otu
- viral
- was\_container

**Next step:** Choose exemplars for tips in phylogenies that are
mapped to higher taxa. See
../exemplified\_phylo/index.html.

**Results:** A taxonomy with a total of 42 nodes.
3 of these are leaves and
39 internal nodes.

The *cleaned\_ott.tre* file in this directory contains a newick
representation of this taxonomy (download newick).

The following table lists the number of OTT Ids pruned by the combination of
flags that caused the pruning. We don't list each of the OTT Ids here because
the list is extremely long and can make your browser hang. The *cleaned\_ott.json*
file in this directory contains the list of pruned Ids
(download cleaned\_ott.json).

| Combination of flags | # of nodes pruned |
| --- | --- |
| barren,inconsistent |

1 |

  |
| empty-after-higher-taxon-tip-prune |

46 |

  |
| higher-taxon-tip |

29 |

  |
| major\_rank\_conflict |

1 |

  |
