# Supplementary material for: Speeding up iterative applications of the BUILD supertree algorithm
Source: PeerJ. 2024 Jan 2;12:e16624. doi: 10.7717/peerj.16624 (PMC10768670; doi:10.7717/peerj.16624)
Supplement: Supplemental Information 7 — A snapshot of otcetera code base with SHA 2287cefc4d12d328c4d9a4272b10aa25b38abbcd from https://github.com/OpenTreeOfLife/otcetera. The algorithms described in this article are implemented in the otc-solve-subproblem tool. [file peerj-12-16624-s007.gz › otcetera/test/tnrs/data/taxonomy-tree/out1.0/grafted_solution/index.html]

Propinquity output documentation. The top-level docs are here.

The *grafted\_solution* directory holds the solution tree created by
grafting together 2 subproblems described in
../subproblem\_solutions/index.html.

The file of interest in this directory is the ./grafted\_solution.tre.

**Next step:** Reattach the taxa that were not present in any
phylogenetic estimate; See
../labelled\_supertree/index.html
