# Supplementary material for: Speeding up iterative applications of the BUILD supertree algorithm
Source: PeerJ. 2024 Jan 2;12:e16624. doi: 10.7717/peerj.16624 (PMC10768670; doi:10.7717/peerj.16624)
Supplement: Supplemental Information 7 — A snapshot of otcetera code base with SHA 2287cefc4d12d328c4d9a4272b10aa25b38abbcd from https://github.com/OpenTreeOfLife/otcetera. The algorithms described in this article are implemented in the otc-solve-subproblem tool. [file peerj-12-16624-s007.gz › otcetera/test/tnrs/data/taxonomy-tree/out1.0/phylo_snapshot/index.html]

Propinquity output documentation. The top-level docs are
here.

The *phylo\_snapshot* directory copies of the NexSON files for each tree that is a
phylogenetic input.

**Next step:** Removing nodes from the phylogenetic inputs
because of problems associated with the mapping of OTUs to the OTTaxonomy.
See ../cleaned\_phylo/index.html
for details about that step.

**Results:**

- concrete\_rank\_collection.json
  is a version of ../phylo\_input/rank\_collection.json
  with git SHAs filled in. Rerunning the pipeline with this collection should
  yield the same inputs.
- collections\_git\_shas.txt
  holds a list of the SHAs for the HEADs of each collection shard of the
  phylesystem at the time the supertree operation was run. In the case of
  this run:
- git\_shas.txt holds a list of the SHAs for the HEADs of each shard of the phylesystem at the time that the supertree operation was run. In the case of this run:
- The 2 NexSON files stored are:
  1. xx\_1@1.json
  2. xx\_2@1.json
