# Supplementary material for: Speeding up iterative applications of the BUILD supertree algorithm
Source: PeerJ. 2024 Jan 2;12:e16624. doi: 10.7717/peerj.16624 (PMC10768670; doi:10.7717/peerj.16624)
Supplement: Supplemental Information 7 — A snapshot of otcetera code base with SHA 2287cefc4d12d328c4d9a4272b10aa25b38abbcd from https://github.com/OpenTreeOfLife/otcetera. The algorithms described in this article are implemented in the otc-solve-subproblem tool. [file peerj-12-16624-s007.gz › otcetera/test/tnrs/data/taxonomy-tree/out1.0/subproblem_solutions/index.html]

Propinquity output documentation. The top-level docs are here.

The *subproblem\_solutions* directory holds the solution to
the 2 subproblems described in
../subproblems/index.html. See
the subproblem\_solutions README
for details on the procedure.

**Next step:** Grafting the subproblems; See
../grafted\_solution/index.html

**Results:** Sorted by the number of tips then by the number
of internal nodes, the subproblem solutions are:

| Subproblem | # tips | # internal nodes |
| --- | --- | --- |
| ott805080.tre | 2 | 1 |
| ott5268475.tre | 2 | 1 |
