# Supplementary material for: Speeding up iterative applications of the BUILD supertree algorithm
Source: PeerJ. 2024 Jan 2;12:e16624. doi: 10.7717/peerj.16624 (PMC10768670; doi:10.7717/peerj.16624)
Supplement: Supplemental Information 7 — A snapshot of otcetera code base with SHA 2287cefc4d12d328c4d9a4272b10aa25b38abbcd from https://github.com/OpenTreeOfLife/otcetera. The algorithms described in this article are implemented in the otc-solve-subproblem tool. [file peerj-12-16624-s007.gz › otcetera/test/tnrs/data/taxonomy-tree/out1.0/labelled_supertree/index.html]

Propinquity output documentation. The top-level docs are here.

The *labelled\_supertree* directory holds the final solution trees,
generated by reattaching the parts of the taxonomy which
were not covered by any input tree (from
../cleaned\_ott/index.html) to the
grafted solution (see
../grafted\_solution/index.html).

**Next step:** Annotate the tree; see
../annotated\_supertree/index.html

**Results**

The input grafted solution had 3 leaves and
2 forking nodes
(and 0 monotypic nodes).

The input cleaned taxonomy had 32 leaves and
20 forking nodes
(and 65 monotypic nodes).

29 leaves were added to create the solution.
0 taxa were rejected.

The files of interest in this directory are (for large files, the link will download the file):

- the ./labelled\_supertree.tre (full solution with names created for non-taxonomic internal nodes).
- the ./labelled\_supertree\_ottnames.tre the same tree with the name\_id format for taxa from OTT.
- the ./broken\_taxa.json which describes who
  members of non-monophyletic taxa are spread across the tree.
- the ./input\_output\_stats.json which describes the
  size of the tree before and after unpruning the taxonomic content.
- the ./labelled\_supertree\_out\_degree\_distribution.txt shows the out-degree frequency distribution
  for the nodes in the labelled supertree
- the ./labelled\_supertree\_ottnames\_without\_monotypic.tre (if the file exists) is a version of labelled\_supertree\_ottnames.tre without any taxa that have only one child.
- the ./labelled\_supertree\_simplified\_ottnames.tre (if the file exists) is a version of labelled\_supertree\_ottnames.tre with names simplified to remove characters that cause problems for some clients (using otc-munge-names). The log for this step will be at simplified\_ottnames.log
- the ./labelled\_supertree\_simplified\_ottnames\_without\_monotypic.tre (if the file exists) is a version of labelled\_supertree\_ottnames\_without\_monotypic.tre with names simplified to remove characters that cause problems for some clients (using otc-munge-names). The log for this step will be at simplified\_ottnames\_without\_monotypic.log

Non-monophyletic taxa: There were 0 non-monophyletic taxa
