# Supplementary material for: Speeding up iterative applications of the BUILD supertree algorithm
Source: PeerJ. 2024 Jan 2;12:e16624. doi: 10.7717/peerj.16624 (PMC10768670; doi:10.7717/peerj.16624)
Supplement: Supplemental Information 7 — A snapshot of otcetera code base with SHA 2287cefc4d12d328c4d9a4272b10aa25b38abbcd from https://github.com/OpenTreeOfLife/otcetera. The algorithms described in this article are implemented in the otc-solve-subproblem tool. [file peerj-12-16624-s007.gz › otcetera/test/tnrs/data/taxonomy-tree/out1.0/exemplified_phylo/index.html]

Propinquity output documentation. The top-level docs are here.

The *exemplified\_phylo* directory holds the results of the last step
of the input preprocessing for the supertree pipeline.
All of the leaves in these trees are mapped to terminal taxa in the taxonomy.
This is accomplished by taking the cleaned input trees (see
../cleaned\_phylo/index.html and
../cleaned\_ott/index.html) and choosing
exemplar taxa to represent at for each tip that is mapped to a non-terminal taxon.
See  for details on the procedure.

**Next step:** Decomposition of inputs into subproblems. See
../subproblems/index.html for details.

**Results:** Below you will find tabular or itemized listings of:

- Exemplified tree files- Map of tree to OTT IDs in that tree that were exemplified- Map of exemplified OTT ID to the IDs used to exemplify it

Exemplified tree files:

- taxonomy.tre -- the version of the taxonomy pruned down to
  only contain the exemplar taxa. This taxonomy has 3 leaves.
- xx\_1@1.tre
- xx\_2@1.tre

The following source input trees were affected by the exemplar step:

| study\_id + tree\_id key | OTT IDs exemplified |
| --- | --- |

The following table shows the exemplars for each of the 0 OTT Ids that were exemplified:

| higher taxon OTT ID | exemplars used |
| --- | --- |
