# Supplementary material for: Speeding up iterative applications of the BUILD supertree algorithm
Source: PeerJ. 2024 Jan 2;12:e16624. doi: 10.7717/peerj.16624 (PMC10768670; doi:10.7717/peerj.16624)
Supplement: Supplemental Information 7 — A snapshot of otcetera code base with SHA 2287cefc4d12d328c4d9a4272b10aa25b38abbcd from https://github.com/OpenTreeOfLife/otcetera. The algorithms described in this article are implemented in the otc-solve-subproblem tool. [file peerj-12-16624-s007.gz › otcetera/test/tnrs/data/taxonomy-tree/out1.0/assessments/index.html]

Propinquity output documentation. The top-level docs are here.

This directory holds the output of
a run of propinquity's `run_assessments.py` script.
That script checks for certain types of problems in the output of the pipeline.
summary.json is a JSON representation of that data
and log.txt is a text log of the assessments run
(the text log will be empty if there are no errors).

The 5 checks run were:

| nickname of check | description |
| --- | --- |
| `lost_taxa` | Check that otcetera's tc-taxonomy-parser and otc-unprune-solution-and-name-unnamed-nodes tools agree about the number of taxa that are not present in the solution |
| `lost_taxa_included_in_tree` | Check that none of the taxa listed as "lost" are in the annotations file |
| `monophyly` | Check that the taxa from the monophyly tests listed in https://github.com/OpenTreeOfLife/germinator/blob/master/taxa/monophyly.csv are monophyletic in the tree. |
| `num_tips` | Check that the cleaned version of the taxonomy and the supertree have the same number of leaves |
| `unsupported_nodes` | Check that none of the nodes listed in the annotations file are completely unsuported |

SUCCESS! No errors were found.
