# Supplementary material for: Speeding up iterative applications of the BUILD supertree algorithm
Source: PeerJ. 2024 Jan 2;12:e16624. doi: 10.7717/peerj.16624 (PMC10768670; doi:10.7717/peerj.16624)
Supplement: Supplemental Information 7 — A snapshot of otcetera code base with SHA 2287cefc4d12d328c4d9a4272b10aa25b38abbcd from https://github.com/OpenTreeOfLife/otcetera. The algorithms described in this article are implemented in the otc-solve-subproblem tool. [file peerj-12-16624-s007.gz › otcetera/doc/full_supertree.pdf]

Thoughts on constructing the Full Supertree

October 23, 2015

In the *otcetera* pipeline, the synthesis tree is first constructed using a pruned taxonomy. This taxonomy has been pruned to remove any leaves that do not occur in one of the ranked input phylogenies. Therefore, a later step in the pipeline involves adding the pruned taxa back in to the synthesis tree. The resulting tree is the *full supertree*. This document is an attempt to collect, and perhaps organize, thoughts on how the full supertree can or should be constructed, as well as related questions and concepts.

This document takes the approach that we should be able to use the sub-problem solver to construct the full supertree by feeding it a sequence of 2 trees:

```
otc-solve-subproblem grafted-solution.tre cleaned_ott.tre
```

Various other quick-and-dirty methods may be sufficient to achieve the result. However, the attempt to make the subproblem solver fast enough to solve this particular problem raises various issues with the subproblem solver. It also suggests various optimizations that may be useful more generally.

1 Potential speed increases

In the current implementation of the BUILD algorithm, we have a number of places we take excessive computation time:

- We attempt to construct rooted splits (a.k.a. desIds) for each node. For a bifurcating tree, this operation should take time and memory quadratic in the number of leaves.
- We attempt to construct connected component by considering each split in a tree separately. However, considering splits for nodes that are not direct children of the root is redundant.
- Much of the time is spent in determining which splits are imposed at a given level in the tree, and therefore need not be passed to subproblems.
  - When different splits have the same leaf set, we should be able to get a speedup.
  - When some splits have the full leaf set, we should be able to get a speedup.
- We recompute connected components from scratch each time BUILD is recursively called on a subproblem. This could be avoided by incrementally removing edges from the graph and discovering new connected components that appear, as in Henzinger et al. *However, it is unclear if an algorithm similar to Henzinger et al could be used to find the edges to remove at each step.*
- When we have two trees T_1 and T_2, and either $\mathcal{L}(T_1) \subseteq \mathcal{L}(T_2)$ or $\mathcal{L}(T_2) \subseteq \mathcal{L}(T_1)$, then it should be possible to determine all conflicting splits in a single pass over the trees, similar to `otc-detectcontested`.

2 The problem

When the subproblem to be solved consists of two ranked trees, T_1 and T_2, and the second tree is the taxonomy, then we have $\mathcal{L}(T_1) \subseteq \mathcal{L}(T_2)$. For each (rooted) split in T_2, we can determine whether that split is consistent with T_1. We claim that each split in T_2 is either consistent with T_1, or incompatible with at least one split of T_1. We can therefore form a new tree T_2' by starting with T_2 and removing each split that is inconsistent with T_1. The splits of T_1 and T_2' are then jointly consistent. Furthermore, by combining the trees T_1 and T_2', we obtain a new tree in which the splits of T_1 that are not implied by T_2' may not fully specify where certain taxa of T_2' are placed. We

resolve this ambiguity by placing such taxa rootward, but their range of attachment extends over specific branches in the tree obtained by combining T_1 and T_2'. All of these branches derive from T_1 but not from T_2'.

First, note that each split of T_2 implies a split on the reduced leaf set $\mathcal{L}(T_1)$. This split then is either consistent with T_1, or conflicts with at least one split in T_1. We obtain T_2' by removing each split of T_2 that, when reducted to leaf set $\mathcal{L}(T_1)$, conflicts with some branch of T_1. By definition, the remaining branches of T_2 are individually compatible with each branch of T_1.

Second, let us look at the subtree of T_2 obtained by restricting the leaf set to $\mathcal{L}(T_1)$. We proceed by adapting a proof that pairwise compatible (unrooted) splits can always be combined to form a tree. The adaptation is necessary because the splits of T_1 and T_2 have different leaf sets. We can handle rootedness in the unrooted context by simply treating the root as a special leaf. Now, let us consider a split σ of T_1 that is not implied by any branch of T_2. Then σ induces a flow from node to node on $T_2|_{\mathcal{L}(T_1)}$ (i.e. T_2 restricted to $\mathcal{L}(T_1)$) by assigning a direction to each branch of $T_2|_{\mathcal{L}(T_1)}$. That is, for each branch of $T_2|_{\mathcal{L}(T_1)}$, σ is either on the left side or the right side of that branch. An important fact (not shown here, but not very hard) is that each node of $T_2|_{\mathcal{L}(T_1)}$ has either all branches pointing towards the node under the flow, or at most one branch pointing away. Therefore, starting from any node, we may follow the flow from node to node along the unique branch directed away from a node. Since this is a flow on a tree, we may not have cycles. Therefore, there must be a fixed point, which occurs when no branches point away from a node. A second important fact (also not shown here) is that when we find a node where all branches point towards to the node, then we may impose the split σ by adding a branch dividing some of the branches at that node from other branches. This completes the proof that if σ is consistent with $T_2|_{\mathcal{L}(T_1)}$ then we may add σ to that tree.

However, in order to add σ to T_2, we must adapt the proof. Specifically, the node of $T_2|_{\mathcal{L}(T_1)}$ where we insert σ is also a node of T_2. However, whereas σ partitions each branch of $T_2|_{\mathcal{L}(T_1)}$ to one side or the other side of the newly inserted branch, there may be branches at the node of T_2 that contain no taxa from T_1. These branches are thus not partitioned to one side or the other side of the new branch. We solve this ambiguity by attaching them to the rootward side of the new branch. However, in reality the branch imposed by σ be part of a connected collection of branches over which these clades of T_2 may wander. Note that not all taxa in $\mathcal{L}(T_2) - \mathcal{L}(T_1)$ may wander across the branch imposed by sigma.

Sigh. Clearly we need some pictures here.

3 Questions

- Is there a single, unique solution to this problem?
- When running BUILD, is it possible to construct a *reason* for the lack of inclusion of each split? Specifically, can we say which split (or set of splits) conflicts with that split?
  - We can construct the graph such that each internal node of a tree is a vertex that connects to the vertex of its children. Instead of cutting branches, we can remove vertices (and their connected edges). (Unlike Stephen's graph, internal nodes from different trees should *not* be merged, I think. At most, we could record that an internal node vertex has multiplicity 2, but each internal node is also going to be associated with a particular leaf set, and it unlikely that the leafs sets would be identical.)
  - If we cut the vertex corresponding to a conflicting split, this should be allow the BUILD algorithm to proceed, *a la* Semple and Steel's mincut supertree method.
